# Study on Self-Humidification in PEMFC with Crossed Flow Channels and an Ultra-Thin Membrane

**DOI:** 10.3390/polym15234589

**Published:** 2023-11-30

**Authors:** Chenlong Wang, Xiaosong Chen, Xin Xiang, Heng Zhang, Zhiping Huang, Xinhao Huang, Zhigang Zhan

**Affiliations:** 1 State Key Laboratory of Advanced Technology for Materials Synthesis and Processing, Wuhan University of Technology, Wuhan 430070, China; 2 Hubei Key Laboratory of Fuel Cells, Wuhan 430070, China

**Keywords:** PEMFC, 3D model, self-humidification, crossed channel, ultra-thin membrane, operating condition, water distribution, cell performance

## Abstract

In this study, a 3D model of a proton exchange membrane fuel cell (PEMFC) with crossed channels and an ultra-thin membrane is developed to investigate the feasibility of self-humidification; experiments utilizing a PEMFC stack with identical configurations are conducted to validate the simulation results and further investigate the effects of various operating conditions (OCs) on self-humidification. The results indicate that the crossed flow channel leads to enhanced uniformity of water distribution, resulting in improved cell performance under low/no humidification conditions. External humidifiers for the anode can be removed since the performance difference is negligible (≤3%) between RHa = 0% and 100%. Self-humidification can be achieved in the stack at 90 °C or below with an appropriate back pressure among 100–200 kPa. As the current density increases, there is a gradual convergence and crossing of the voltage at low RH with that at high RH, and the crossover points are observed at 60–80 °C with suitable pressure when successful self-humidification is achieved. Below the current density of the point, the stack’s performance is inferior at lower RH due to membrane unsaturation, and conversely, the performance is inferior at higher RH due to flooding; this current density decreases with higher pressure and lower temperature.

## 1. Introduction 

Proton exchange membrane fuel cells (PEMFCs), as promising energy conversion devices with higher power density, lower noise, and zero pollution, have received great attention [1,2,3]. Despite its many advantages, there are still many technical challenges that need to be solved to achieve efficient power supply [4]. The main issue that limits the performance of the PEMFC is the ohmic loss, which is mainly caused by the resistance of the proton exchange membrane (PEM) especially when it has lower water content [5]. Therefore, water management is of vital importance to achieve maximum performance and durability from PEMFCs [6]. An external humidification system is usually added to provide hydration for the PEM. However, it not only increases the complexity of the vehicle system structure but also requires an additional power supply, which in turn reduces the net output power [7]. Therefore, it is essential to remove the external humidification system and achieve self-humidification of the PEMFC [8].

There has been extensive research on how to improve the performance of PEMFCs at low/no humidification [9], including the alternation of the gas diffusion layer (GDL) [10,11], microporous layer (MPL) [12], catalyst layer (CL) [13,14], and PEM [15,16]. To achieve self-humidification of PEMFC, the priority is to enhance its low-humidity performance. Some research focuses on improving water production, retention, and transport in the membrane electrode assembly (MEA). For example, Cha et al. [17] conducted experiments to measure the performance of self-humidifying PEMFCs with short-side-chain (SSC) and long-side-chain (LSC) membranes. They found that the power density of the self-humidifying PEMFC with the SSC membrane was higher than that with the LSC membrane due to increased water retention. Li et al. [18] performed a three-dimensional modeling of PEMFC. They found that compared to the thick catalyst-coated membrane (CCM), the thin CCM is more easily hydrated by water generated by reaction under low humidity conditions. Huang et al. [19] designed and fabricated a novel composite PEM based on vinyl-phosphonic acid-functionalized mesoporous silica nanoparticles. They found that the VPA-MSN(–NH_2_) endows the composite membrane with high water uptake and water retention, enhances proton conductivity, and improves cell performance under low relative humidity (RH) conditions. Xie et al. [20] enhanced the low-humidity performance of PEMFC by incorporating a phosphoric acid-loaded covalent organic framework in the anode catalyst layer. Shin et al. [21] developed a two-phase PEMFC model to predict the performance under both dry and humid conditions. They found that the high tortuosity of the CL can support the prevention of anode dehydration under lower humidity conditions by accelerating the back diffusion of water from the cathode to the anode side. Hou et al. [22] enhanced the low-humidity performance of PEMFC by introducing hydrophilic carbon nanotubes in the MEA. Angayarkanni et al. [23] coated a silica composite layer on the Pt/C CL and prepared a MEA that delivers excellent fuel cell performance in dry gas conditions. Ren et al. [24] prepared a nanostructured GDL with enhanced hydrophobicity to improve the water management performance of the PEMFC. They found that the GDL can improve capillary pressure to help retain liquid water at the GDL/CL interface and facilitate the wetting of the membrane under low humidity conditions. Li et al. [25] based their study on the introduction of graphene oxide polymer brushes as an inorganic additive and incorporated Pt-TiO_2_ nanoparticles fixed on a polymeric PEM to form a cross-linked network structure. This allowed them to prepare novel cross-linked PEMs and nanocomposite PEMs with high self-humidification performance.

In addition, the structure of the flow field also greatly influences water management in achieving self-humidification. Tong et al. [26] designed a cross-convection flow channel and investigated the distribution of water heat and its performance at various positions. They found that the uniform distribution of water in the cross-convection channel prevents the phenomenon of membrane drying inside the PEMFC. Wang et al. [27] developed a quasi-three-dimensional transient non-isothermal model to explain various experimental phenomena. They found that, compared with co-flow configurations, counter-flow configurations improve the uniform distribution of water and current density and reduce the probability of flooding in segments near the outlet gas channel. Zhang et al. [28] investigated the dynamics of droplet behavior in different channel sizes within the micro-flow field and examined their impact on PEMFC performance. They found that the PEMFC with the micro-flow field can achieve the most excellent performance without air humidification. Lian et al. [29] proposed a novel porous flow field that utilizes metal fibers to create a network of continuous capillary pathways. The experimental results show that the fiber flow field exhibits optimal performance at 50 °C under low humidification, and an increase in back pressure can significantly enhance the performance of the fuel cell. Additionally, they optimized the structure to further improve the peak power density and the average output current of the fiber porous self-humidifying flow field [30].

Furthermore, recent studies have also found that self-humidification through anode/cathode circulation can replace the use of external humidifiers. Fan et al. [31] developed a three-dimensional multiphase numerical model for PEMFC. The results show that the water produced at a high current density in the cathode CL is sufficient to humidify the polymer electrolyte by flowing back and forth between the anode and cathode. Zhao et al. [32] experimentally investigated the dynamic performance and stability characteristics of a fuel cell system with dual exhaust gas recirculation using an orthogonal test method. They found that with dual recirculation, the fuel cell membrane can be well hydrated, and the system performance only shows a 3% reduction compared to a system with an external humidifier. Shao et al. [33] proposed a dynamic model for a PEM fuel cell system that includes anodic and cathodic exhaust gas recirculation. The results showed that the humidification effect is much better for cathode recirculation compared to anode recirculation, especially under low inlet RH conditions. Zhang et al. [34] constructed a test bench for a hydrogen fuel cell system with a recycling cathode. The experimental results demonstrate that the cathode recirculation can prevent membrane dryness at low current density without external humidifiers and improve the water removal capability of the stack outlet at high power. Zhang et al. [35] established a dynamic mechanism model for a control-oriented hydrogen fuel cell system based on cathode exhaust gas recirculation. They found that at high current densities, increasing the operating temperature can reduce the saturation of liquid water on the cathode side and prevent fuel cell flooding. Liu et al. [36] developed a comprehensive dynamic control model for an automotive self-humidifying fuel cell system with cathode recirculation. The results show that the control model can suppress high potential and increase the cathode RH, which is significant in solving the durability decay caused by low humidity under low load conditions of self-humidifying systems. Moreover, bipolar membrane fuel cells are also used to achieve self-humidification. Li et al. [37] established a two-dimensional steady-state MEA model of bipolar membrane fuel cells to investigate the water transport mechanism and the formation of self-humidification in this novel MEA structure. They found that the thickness of the anion exchange membrane (AEM) affects the effective diffusion of water, and the variation in the membrane’s water uptake property can also influence the intrinsic concentration gradient of water in the membrane. Heo et al. [38] prepared a fuel cell (FC) by laminating the PEM and AEM composed of acceptor-doped SnP2O7 composites. The self-humidifying fuel cell membrane demonstrates exceptional electrochemical performances under harsh operating conditions (OCs) of high temperature and low RH. Wang et al. [39] proposed a self-humidification design that utilizes a series connection of the PEM and AEM fuel cells. They found that the performance can be improved by reducing the thickness of the AEM and the flow rate of the anode through simulation.

In summary, PEMFC humidification has been an important and interesting topic. Although there are numerous studies on self-humidification by modeling, most of them are only validated with simple experiments. In our study, experimental and modeling research are both conducted on the self-humidification of FC together, especially the experimental operation of a stack that is very close to an industry application. Meanwhile, the stack has no anode or cathode gas circulation system or AEM, and the uniformity of water distribution is considered by a specially designed flow field with crossed channels for the anode and cathode flow field plates. Therefore, a 3D model of FC with a novel crossed channel structure and an ultra-thin membrane of 8 μm is developed to investigate the feasibility of self-humidification; experiments utilizing a PEMFC stack with identical configurations are conducted to validate the simulation results and further investigate the effects of various OCs on self-humidification. The following sections contain model development, numerical methodology, experimental equipment and methods, results and discussion, and conclusions.

## 2. Model Descriptions

### 2.1. Geometric Model

**Figure 1:**
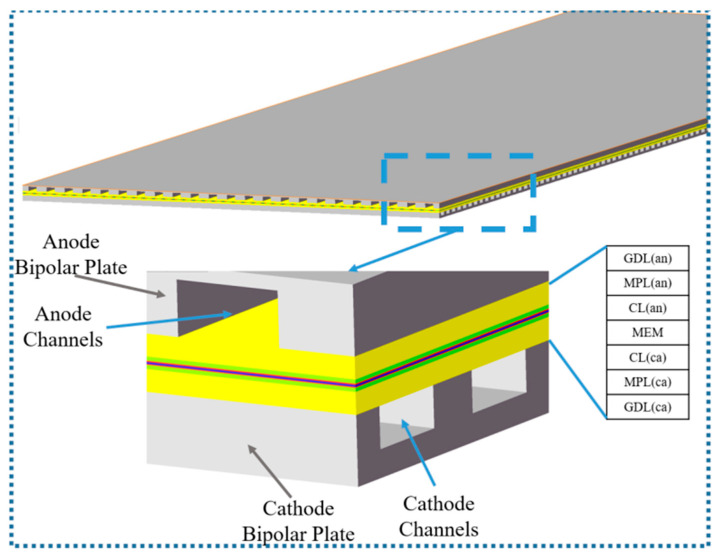
Schematic of the PEMFC model.

In this work, a 3-dimensional PEMFC steady-state model with crossed flow channels is developed. The geometric domain is shown in Figure 1. The geometric model consists of the following components: anode/cathode bipolar plate (BP), anode/cathode flow channel, anode/cathode GDL, anode/cathode MPL, anode/cathode CL, and MEM. The overall dimensions of the single cell are 120.1 mm × 41.6 mm × 1.407 mm, and each component’s dimensions are listed in Table 1.

### 2.2. Model Assumptions

To simplify the simulation model, the following assumptions are made:

(1) The flow in the flow channel is regarded as laminar flow [40];
(2) The reaction gases are ideal and incompressible;
(3) The water generated by the reaction in the CL is membrane water [41];
(4) The porous media is assumed to be isotropic and uniform;
(5) The crossover of the hydrogen permeation phenomenon in MEM is ignored [42];
(6) Ignoring the influence of gravity.

### 2.3. Governing Equations

The multi-physics field in this model involves heat transfer, mass transfer, and electrochemical reactions. Under the aforementioned assumptions, the governing equations in this three-dimensional and steady-state model are presented as follows.

**Mass conservation equation:**

(1) $$\nabla •(\rho \varepsilon \vec{u})=S_{m}$$

where ρ is the density of the fluid, ε is the porosity of the porous media, $\vec{u}$ is the fluid velocity, $S_{m}$ is the source term.

**Species conservation equation:**

(2) $$\nabla \cdot \left(\varepsilon \vec{u}c_{i}\right)=\nabla \cdot \left(D_{i}^{\mathrm{eff}}\nabla c_{i}\right)+S_{i}$$

where $c_{i}$ is the molar fraction, $D_{i}^{eff}$ is the multi-component diffusivity, and $S_{i}$ is the source term.

**Momentum conservation equation:**

(3) $$\nabla •(\varepsilon \rho \vec{u}\vec{u})=-\varepsilon \nabla p+\nabla •(\varepsilon \mu \nabla \vec{u})+S_{u}$$

where μ is viscosity, p represents pressure, $S_{u}$ is the source term.

**Energy conservation equation:**

(4) $$\nabla •\left[\sum_{i=g,l}^{}{(\varepsilon \rho c_{p}\vec{u})}_{i}T\right]-\nabla •(\sum_{i=g,l,s}^{}k_{i}^{eff}\nabla T)=S_{Q}$$

where $c_{p}$, T, $k^{eff}$ represent constant pressure specific heat, temperature, and thermal conductivity, respectively. $S_{Q}$ is the source term.

**Charge conservation equation:**

In this model, the Butler–Volmer equations for the anode and cathode are used to calculate the reaction rate in CL of anode and cathode:

(5) $$\nabla •\left(k_{ele}\nabla \varphi_{ele}\right)+S_{ele}=0$$

(6) $$\nabla •\left(k_{ion}\nabla \varphi_{ion}\right)+S_{ion}=0$$

where $k_{\mathrm{ele}}$, $k_{ion}$, $\varphi_{ele}$, $\varphi_{ion}$, $S_{\mathrm{ele}}$, $S_{ion}$ represent electron conductivity, proton conductivity, solid phase potential, membrane potential, electron source term, and proton source term, respectively.

The description of the details of all the source terms can be referred to in [43,44].

### 2.4. Water Transport

In a PEMFC, variations in materials and structure can result in various states and phase change behavior of the water. Typically, the state and phase of water vary within the membrane, CL, GDL, and channels.

**Membrane water equation:**

(7) $$-\nabla \cdot (\frac{n_{d}}{F}k_{ion}\nabla \phi_{ion})=\nabla \cdot (\frac{\rho_{m}}{EW}D_{\lambda }\nabla \lambda )+S_{\lambda }+S_{\mathrm{gd}}+S_{\mathrm{ld}}$$

where $n_{d}$ is the electro-osmotic drag coefficient, $\rho_{m}$ is the membrane density, $D_{\lambda }$ is the water diffusivity, EW is the equivalent mass of PEM, λ is the water content. $S_{\lambda }$ is the water generation rate due to the cathode side reaction in the catalyst layer, $S_{\mathrm{gd}}$ is the rate of mass change between gas and dissolved phase, and $S_{\mathrm{ld}}$ is the rate of mass change between liquid and dissolved phase.

(8) $$n_{d}=2.5\frac{\lambda }{22}\;(0⩽\lambda ⩽22)$$

(9) $$D_{\lambda }=D_{\lambda }^{ref}\cdot e^{2416(\frac{1}{303}-\frac{1}{T})}$$

(10) $$D_{\lambda }^{ref}=\left\{\begin{array}{ll} 3.1\cdot 10^{-7}\lambda \left(e^{0.28}-1\right)e^{\frac{-2346}{T}}, & \lambda <3\\ 4.17\cdot 10^{-8}\lambda \left(161e^{-\lambda }+1\right)e^{\frac{-2346}{T}}, & \lambda \geq 3 \end{array}\right.$$

$S_{gd}$ and $S_{ld}$ are calculated by:

(11) $$S_{\mathrm{gd}}=\left(1-s^{\theta }\right)\gamma_{\mathrm{gd}}M_{H_{2}O}\frac{\rho_{m}}{EW}\left(\lambda_{\mathrm{eq}}-\lambda \right)$$

(12) $$S_{\mathrm{ld}}=s^{\theta }\gamma_{\mathrm{ld}}M_{H_{2}O}\frac{\rho_{m}}{EW}\left(\lambda_{\mathrm{eq}}-\lambda \right)$$

where s is the liquid saturation, $\gamma_{gd}$ and $\gamma_{ld}$ is the gas and liquid mass exchange rate constants, $\lambda_{\mathrm{eq}}$ is the equilibrium water content.

**Liquid water transport equation in porous media:**

(13) $$\nabla \cdot \left(\frac{\rho_{l}\kappa \kappa_{r}}{\mu_{l}}\nabla p_{l}\right)+S_{\mathrm{gl}}-S_{\mathrm{ld}}=0$$

where $\rho_{l}$ is the liquid water density, κ is the absolute permeability, $\kappa_{r}$ is the relative permeability, $S_{\mathrm{gl}}$ is the conversion rate of gaseous and liquid water and $S_{\mathrm{ld}}$ is the conversion rate of liquid and membrane water.

$S_{\mathrm{gl}}$ is calculated by:

(14) $$S_{\mathrm{gl}}=\left\{\begin{array}{ll} \gamma_{e}\varepsilon sD_{\mathrm{gl}}\frac{M_{w}}{RT}p\ln\left(\frac{p-p_{\mathrm{sat}}}{p-p_{\mathrm{wv}}}\right), & p_{\mathrm{wv}}\leq p_{\mathrm{sat}}\\ \gamma_{c}\varepsilon (1-s)D_{\mathrm{gl}}\frac{M_{w}}{RT}p\ln\left(\frac{p-p_{\mathrm{sat}}}{p-p_{\mathrm{wv}}}\right), & p_{\mathrm{wv}}>p_{\mathrm{sat}} \end{array}\right.$$

where $\gamma_{e}$ is the evaporation rate coefficient, $p_{\mathrm{wv}}$ is the water vapor pressure, $\gamma_{c}$ is the condensation rate coefficient, $p_{\mathrm{sat}}$ is the saturated vapor pressure. 

$D_{\mathrm{gl}}$ is a coefficient and calculated by:

(15) $$D_{\mathrm{gl}}=\left\{\begin{array}{l} 0.365\cdot 10^{-4}{\left(\frac{T}{343}\right)}^{2.334}\left(\frac{10^{5}}{p}\right)\;\mathrm{Cathode}\\ 1.79\cdot 10^{-4}{\left(\frac{T}{343}\right)}^{2.334}\left(\frac{10^{5}}{p}\right)\;\;\;\;\;\;\mathrm{Anode} \end{array}\right.$$

**Liquid water transport equation in channels:**

(16) $$\nabla \cdot \left(\rho_{l}\vec{u_{l}}s\right)=\nabla \cdot \left(D_{\mathrm{liq}}\nabla s\right)$$

(17) $${\vec{u}}_{l}=\chi {\vec{u}}_{g}$$

where $D_{\mathrm{liq}}$ is the diffusion coefficient of liquid water in the gas channels, ${\vec{u}}_{l}$ is the velocity of liquid water, ${\vec{u}}_{g}$ is the velocity of gaseous water, χ is the velocity ratio of liquid water to gaseous water in the flow channel.

**The conductivity of the membrane**

In addition, the conductivity of the membrane is related to the membrane water content, and can be calculated by:

(18) $$\sigma \left(T,\lambda \right)=\sigma_{303K}\left(\lambda \right)\exp\left[1268\left(\frac{1}{303}-\frac{1}{T}\right)\right]$$

(19) $$\sigma_{303K}\left(\lambda \right)=0.005193\lambda -0.00326$$

### 2.5. Numerical Methodology and Boundary Conditions

In this study, the governing equations described earlier are solved using double precision, employing the finite volume method. The computational fluid dynamics (CFD) software Ansys Fluent 2022 R1 is employed to solve the model. The cell inlet is set as the mass flow inflow, and the outlet as the pressure boundary. The temperature of the wall is set as constant, and the constant current control mode is adopted for the calculation. The boundary conditions and electrochemical parameters are listed in Table 2.

### 2.6. Model Verification

**Figure 2:**
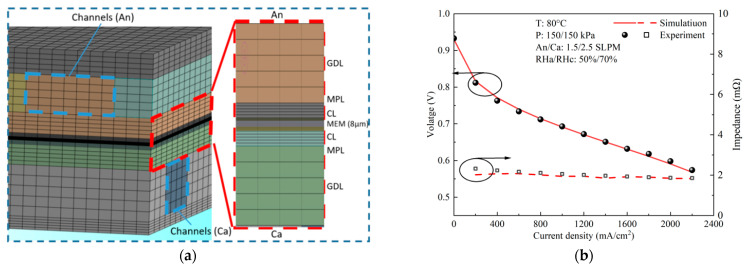
(**a**) The mesh model; (**b**) polarization curve by experiment and modeling.

The mesh model is shown in Figure 2a, and the grid independence test is carried out. Table 3 shows the calculation results and time of different grid quantities for the cases with the same boundary conditions. It can be found that Case 2 can effectively balance computational efficiency and computational accuracy. Therefore, 10,991,552 grids are selected to calculate the model. Figure 2b shows the comparison of simulated and tested voltage and impedance under the same OCs, and the circles and arrows indicates that the corresponding curves and what they represents. It is observed that the experimental results fit well with the simulation data, which verifies the validity of the simulation model.

## 3. Experiment

### 3.1. Experimental Equipment

**Figure 3:**
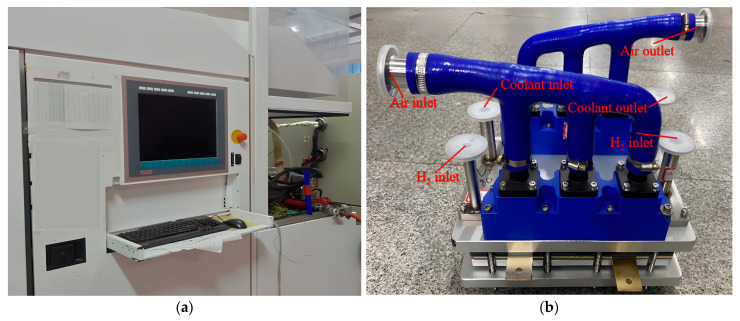
(**a**) The test system; (**b**) the fuel cell stack.

Figure 3a is the self-humidification test system, which consists of a test bench of Greenlight G500, the FC stack, a high-frequency impedance (HFR) meter, and other necessary equipment. The G500 is used for feeding reactants (air and H_2_) and coolant, temperature, humidity, load regulation, and control. The HFR meter from KIKUSUI company (Yokohama, Japan) (KFM2151) ranges from 0.001 mΩ to 33 kΩ with an accuracy of 0.2%. The FC stack (shown in Figure 3b) is provided by WUT New Energy Co., Ltd. (Wuhan, China), composed of 10 single cells, end plates, insulating plates, and current collector plates. In each cell, the crossed flow channel is arranged in the bipolar plates, the PEM is Gore-SELECT^®^ M765.08 with a thickness of 8 μm, the CL is 60% platinum carbon catalyst, with E-type carbon as the carriers, and Pt loading was 0.4 mg/cm^2^ for the cathode and 0.1 mg/cm^2^ for the anode. The GDL is from Toray Company (XGL-30T, hydrophobicity of 10%), Tokoyo, Japan.

### 3.2. Experimental Methods

To investigate the effects of different OCs on self-humidification, experiments are conducted under various current densities, pressures, temperatures, and RH. The tested regimes are given as follows: (1) The stack is activated for 2 h to achieve an optimal working state. (2) The current density is increased from 0 to 2000 mA·cm^−2^ or higher, with a step size of 100 mA·cm^−2^. (3) The running time for each current density is no less than 5 min and then the voltage and impedance are recorded. (4) Once one test is finished, the stack will be purged, and then the test under other OCs is carried out. All OCs are given in Table 4.

It is very difficult to directly measure the water content in the membranes of a FC stack. However, for a certain FC, the impedance is directly correlated to the hydration state of the membrane, and the performance is directly influenced by the impedance. Therefore, during the experiment, the impedance is measured by the HFR meter at a frequency of 1 kHz, which is considered equivalent to the ohmic resistance [29,30], to indicate the membrane water content, so as to judge if the FC self-hydrates.

## 4. Results and Discussion

### 4.1. Effects of the Crossed Channel on Water Distribution

**Figure 4:**
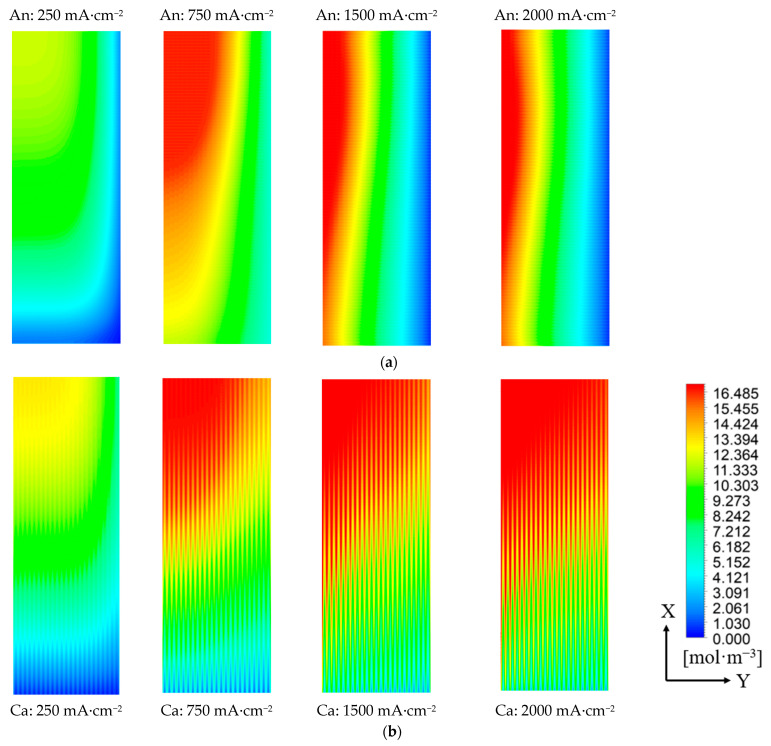

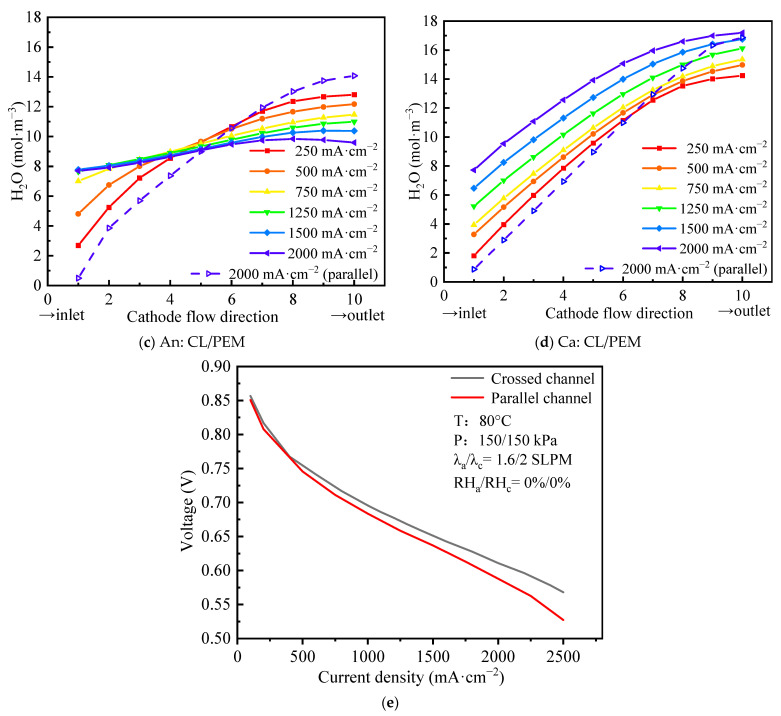
Water molar concentration on the interface of CL/GDL (**a**) at the anode and (**b**) at the cathode; water molar concentration along the X direction on the interface of CL/PEM (**c**) at the anode and (**d**) at the cathode; (**e**) cell performance by modeling between crossed and parallel channels.

Figure 4 shows the modeled results in the cell with crossed channels and an ultra-thin membrane of 8 μm. The FC operates at a temperature of 80 °C, a back pressure of P = 150/150 kPa, a stoichiometric ratio of λ_a_/λ_c_ = 1.6/2, and RHa/RHc = 0%/0%. In Figure 4a,b, the distribution of the water molar concentration at the interface of CL/GDL at different current densities (250/750/1500/2000 mA·cm^−2^) is presented, and the air flows along the X-direction, while the hydrogen flows against the Y-direction. The distribution of water on the anode side shows an obvious step change in the Y-direction, which is determined by the flow of dry H_2_. The concentration increases more smoothly on the cathode side. As the current density increases, the water on both the cathode and anode sides generally increases, and the distribution becomes more uniform.

In Figure 4c,d, the displayed water molar concentration on the interface of CL/PEM is the average value of 10 separate areas along the X direction. When the current density increases, the water on the interface of CL/PEM at the anode becomes more uniform, and the deviation between the inlet and outlet shrinks gradually, while at the cathode, the water increases incrementally along the X direction. Additionally, the molar concentration of water in a parallel channel, counter-flow configuration cell at 2000 mA·cm^−2^ is also given. At the anode, the water molar concentration gradient in the parallel channel cell at 2000 mA·cm^−2^ is higher than that in the crossed channel cell at all current densities. At the cathode, the molar concentration of water in the parallel channel cell is obviously lower than that in the crossed channel cell. Figure 4e shows the cell performance by modeling between the cell with crossed and parallel channels (counter flow). When the current density increases, the performance difference between the cells with crossed and parallel channels increases, ultimately reaching a maximum of 0.041 V (7.2%) at 2500 mA·cm^−2^.

In the crossed channel cell, the uniformity of water distribution is improved on the anode side, and the amount of water on the cathode side is increased compared to the parallel channel counter-flow configuration cell when operated under no humidity conditions. Since the uniformity of water distribution is improved, it is beneficial to saturate the PEM and strengthen its proton conductivity, resulting in an increased electrochemical reaction rate and water molar concentration at the cathode. Therefore, the crossed channel can achieve better cell performance without any humidifiers compared to the parallel channel cell due to more water being preserved in the MEA. Meanwhile, increasing the current density can also improve the water molar concentration and the uniformity of its distribution on both sides, which enhances the electro-osmotic drag and is beneficial to achieve the self-humidification of FC.

### 4.2. Effects of RHa on Cell Performance 

**Figure 5:**
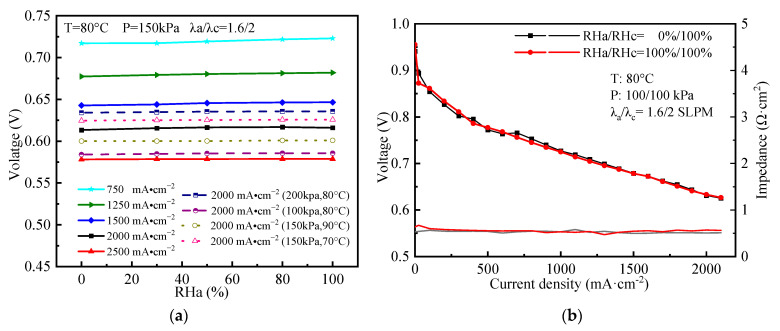
(**a**) Cell performance by modeling at different OCs; (**b**) stack performance by experiments at RHa = 0/100%, RHc = 100%.

Figure 5a shows the cell performance at different OCs by modeling. It can be found that the cell voltage changes are 5.9/4.48/3.74/2.7/0.53 mV between RHa = 0% and RHa = 100% at the current density of 750/1250/1500/2000/2500 mA·cm^−2^, respectively, when the temperature is 80 °C and the back pressure is 150/150 kPa. Meanwhile, at different temperatures and pressures, the cell performance also varies little when the RHa changes. In Figure 5b, the voltage and impedance of the stack with crossed channels and an ultra-thin membrane are tested at P = 100/100 kPa, λ_a_/λ_c_ = 1.6/2, 80 °C, and RHa = 0/100%, RHc = 100%. It can be observed that there is a negligible difference in the stack voltage/impedance between the case with and without anode humidification. The measured difference is less than 1% at ≥800 mA·cm^−2^, and ≤3% at all current densities. Therefore, the tested stack performance effectively validates the simulation results.

The water transported across the membrane by electro-osmotic drag and back diffusion saturates the membrane [33,45], which can be effectively improved by the ultra-thin membrane, resulting in FC performance insensitive to the change of RHa under most OCs. When the voltage difference of the stack varies by less than 3% at all current densities between low/no and high humidification conditions [32,46], the self-humidification is considered to be successful. Consequently, due to anode insensitivity to the change of RHa and the measured data shown in Figure 5, it is concluded that the anode can effectively achieve self-humidification, and the anode external humidifier can be removed for the PEMFC with crossed channels and an ultra-thin membrane.

### 4.3. Effects of Pressure on Self-Humidification

**Figure 6:**
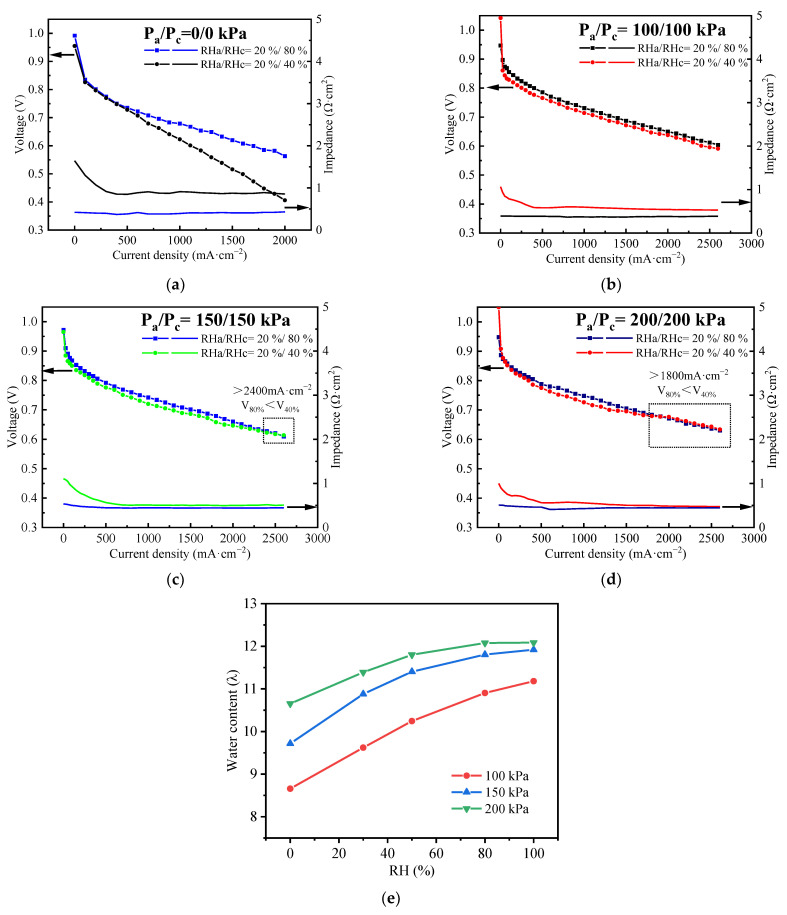
(**a**–**d**) Performance curve of the stack at 80 °C with different back pressure: 0/100/150/200 kPa by experiments, respectively. (**e**) Water content in the membrane at different back pressures: 0/100/150/200 kPa by modeling.

Figure 6a–d shows the comparison of experimental data of stack voltage and impedance at four different back pressures: 0/100/150/200 kPa. The other OCs are: T = 80 °C, λ_a_/λ_c_ = 1.6/2, RHa/RHc = 20%/80%, and 20%/40%, respectively. Figure 6e shows the water content in the membrane at different back pressures: 0/100/150/200 kPa by modeling. The arrows indicates what the curves represent.

Figure 6 clearly shows that the performance of the stack improves as the back pressure increases. The impedance of the stack remains almost constant and low in all four cases for all current densities at RHa/RHc = 20%/80%, but it is relatively high at RHa/RHc = 20%/40% below 500 mA·cm^−2^. As the current density increases, it gradually decreases and remains almost constant after reaching 500 mA·cm^−2^. This indicates that even in the FC with an ultra-thin membrane of 8 μm, the membrane fails to attain adequate hydration when operated at low RH and a current density below 500 mA·cm^−2^. Figure 6a shows the I–V curves of 0 kPa back pressure. The voltage difference rises with the increase in current density because the impedance remains much higher for the case of RHa/RHc = 20%/40%, even after 500 mA·cm^−2^. The impedance difference between the two RHs is quite large, which amplifies the voltage difference along with the increase in current density. It can be observed that even at 2000 mA·cm^−2^, there is a voltage and impedance difference of 0.157 V (28%) and 0.42 mΩ·cm^2^ between RHa/RHc = 20%/80% and 20%/40%, respectively. It is demonstrated that the self-humidification fails when the back pressure is at 0 kPa.

As the back pressure increases, the drainage of water from the MEA to the gas channels is hindered, leading to a higher retention of water in the MEA [30,46,47]. Consequently, the membrane becomes more hydrated, causing weakened effectiveness of external humidification, resulting in a reduction in the impedance differences between high/low humidification conditions, as shown in Figure 6b–d. 

Figure 6b shows the I–V curves of 100 kPa back pressure. The disparity in performance and impedance between the two RHc diminishes as the current density increases. Specifically, at a current density of 2000 mA·cm^−2^, the differences are measured to be 0.012 V (1.8%) and 0.143 mΩ·cm^2^ at 2000 mA·cm^−2^, respectively. As the pressure increases to 150 kPa (Figure 6c), the two performance curves at different RH cross at 2400 mA·cm^−2^, which is because when the current density of the stack at low RH is low, the water generated by the reaction is insufficient, so there is inadequate water content in the PEM, leading to a lower performance than that at high RH. However, when self-humidification is successfully achieved, water flooding may occur at high current density and higher RH, which would obstruct the transportation of the reactant gases, resulting in lower performance than that at lower RH. Therefore, the two performance curves of different humidification cross at a specific current density and there is a crossover point. As the pressure increases to 200 kPa (Figure 6d), the current density of the crossover point diminishes to 1800 mA·cm^−2^, obviously lower than 2400 mA·cm^−2^ at a back pressure of 150 kPa. In Figure 6e, the water content in the membrane relative to RH at 2000 mA·cm^−2^ is plotted by modeling at different pressures. It is observed that increasing the back pressure can improve the water content, and the higher the back pressure, the lower the gradient of water content changes with RH. This is attributed to the fact that as the back pressure increases, water becomes more difficult to evaporate, and the pressurization impedes the drainage of water from the GDL to the gas channels, resulting in increased water content in the membrane, improving the water diffusivity and the proton conductivity of the membrane, reducing the impedance and improving the cell performance at low RH, and causing the crossover point to appear earlier at higher back pressure, as shown in Figure 6c,d.

Hence, it is beneficial to achieve self-humidification by elevating the back pressure. For fuel cells operating at 80 °C without or with low external humidification, it is necessary to maintain a back pressure of at least 100 kPa.

### 4.4. Effects of Temperature on Self-Humidification

**Figure 7:**
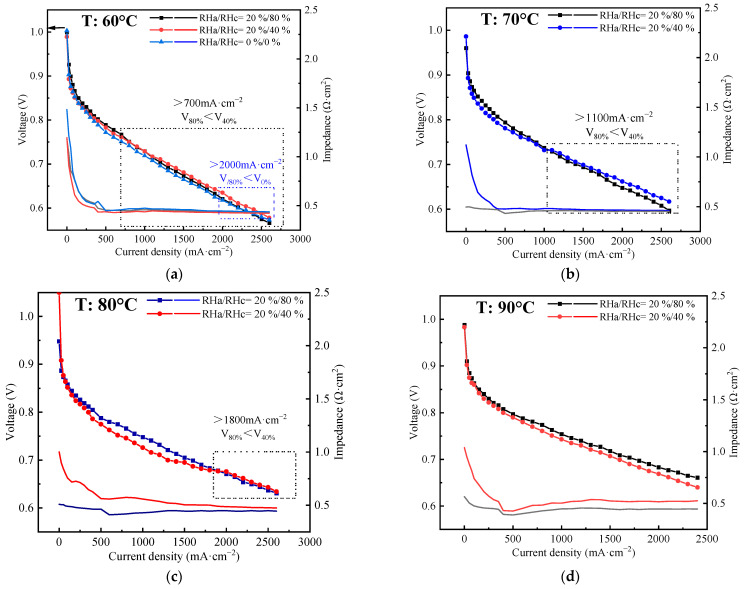
(**a**–**d**) Polarization curve of the stack at 80 °C with a back pressure of 200 kPa at different temperatures: 60/70/80/90 °C by experiments, respectively.

Temperature is an important factor affecting the working efficiency and durability of PEM fuel cells whose typical operating temperature range is between 60 °C and 90 °C [48]. Therefore, the effects of temperature on self-humidification were investigated. Figure 7a–d presents a comparison of the experimental data of the stack voltage and impedance at four distinct temperatures: 60 °C/70 °C/80 °C/90 °C. For each case, the other OCs are as follows: P = 200/200 kPa, λ_a_/λ_c_ = 1.6/2.

Similar to the case at 80 °C, the I–V curves depicted in Figure 7a,b at the high/low RH gradually converge and cross at a specific point as the current density rises. Notably, the current density at the point is lower for the case with a lower temperature. Meanwhile, the variation in impedance difference between RHa/RHc = 20%/80% and 20%/40% exhibits an upward trend as the temperature rises. At 2000 mA·cm^−2^, the impedance gap between RHc = 80%/40% is measured to be 0.008 mΩ·cm^2^ at 60 °C and 0.009 mΩ·cm^2^ at 70 °C, respectively. Furthermore, Figure 7a presents the I–V data for RHa/RHc = 0% at a temperature of 60 °C. It can be observed that the maximum voltage loss for RH = 0% is 0.023 V (2.15%) at 500 mA·cm^−2^, and it crosses with the curve of RHa/RHc = 20%/80% at 2000 mA·cm^−2^; the impedance at different RH remains relatively consistent. In other words, achieving self-humidification at a temperature of 60 °C is relatively straightforward. Figure 7d illustrates the performance at 90 °C between RHc = 40% and 80%, and the maximum deviations in voltage and impedance are observed to be 0.019 V (2.88%) and 0.079 mΩ·cm^2^, respectively. According to the definition of self-humidification provided in Section 4.2, it can be concluded that self-humidification at 90 °C is successful, but there is no crossover between the two curves in the measured data. Meanwhile, the performance of the stack at 90 °C surpasses that at 80 °C due to the enhanced reaction rate.

It can be observed that as the temperature increases, the performance curves cross at a higher current density, because the sulfonate groups tend to lose more water, which ultimately results in reduced water content in the PEM and an increase in stack impedance. Therefore, it is more difficult for the stack to achieve self-humidification at higher temperatures, especially at 90 °C. However, increasing the back pressure can compensate for the negative effect of increasing temperature, as outlined in Section 4.3. As a consequence, self-humidification at low RH can be successfully accomplished even at a high temperature of 90 °C with a back pressure of 200/200 kPa. Furthermore, it has been observed that increasing the temperature within the range of 60–90 °C can lead to enhancements in both the drag coefficient and membrane conductivity if the water content is kept constant [49], and the water quantity and its distribution can be improved to a relatively high and stable level in the crossed arranged channel cell and ultra-thin membrane; the catalyst activity is also promoted by increasing temperature. Therefore, the performance of the stack is enhanced as the temperature increases within the range of 60–90 °C.

In conclusion, when operated within the temperature range of 60–90 °C and under a back pressure of 200 kPa, the FC stack is capable of achieving self-humidification.

### 4.5. Crossover Points

As observed in Figure 6 and Figure 7, there is a crossover point where the performance of the stack at RHa/RHc = 20%/40% is equal to that at RHa/RHc = 20%/80%. Before reaching the current density of the crossover point, the performance of the stack at low RH is inferior to that at high RH. After this current density, the voltage of the stack at high RH is lower than that at low RH. In Table 5, the crossover points at different OCs are presented, revealing that the crossover point only appears when self-humidification is successfully achieved. Furthermore, the current density of the crossover point tends to decrease with lower temperature and higher pressure. In addition, it is observed that the crossover point of RHa/RHc = 0% appears at 2000 mA·cm^−2^, 60 °C, and 200 kPa.

When the current density of the stack at low/no humidification is low, the water generated by the reaction is insufficient, so there is inadequate water content in the PEM, leading to a lowered performance of the stack. However, when self-humidification is successfully achieved, water flooding may occur at high current density and humidification, which would obstruct the transportation of the reactant gases, resulting in a decrease in stack performance [50]. Therefore, the two performance curves of different humidification cross. Meanwhile, at low temperatures or high pressure, self-humidification is easier to achieve, and water flooding is more prone to happen in the stack at high humidification. Therefore, the current density of the crossover points tends to decrease at lower temperatures or higher pressures.

## 5. Conclusions

In this study, a three-dimensional steady-state PEMFC model with crossed channels and an ultra-thin membrane of 8 μm is developed to investigate the feasibility of self-humidification. Meanwhile, the self-humidification experiments are conducted using a PEMFC stack with the same configuration under various pressures and temperatures. The main conclusions can be summarized as follows:

(1) When operated under no humidity conditions, for the FC with crossed channels and an ultra-thin membrane, the uniformity of water distribution is improved, and the water concentration is increased compared with the parallel channel counter-flow configuration cell. Therefore, the cell can achieve better performance without humidifiers than the parallel channel cell. Meanwhile, increasing the current density can also improve the water concentration and the uniformity of water distribution.
(2) The anode is insensitive to the change of RHa under most OCs because the anode water is mainly transported from the cathode by back diffusion, which can be improved by the ultra-thin membrane. This is verified by the measured performance of the stack operated at Pa/Pc = 100/100 kPa, T = 80 °C, and RHc = 100%, and the performance difference between with and without anode humidification can be ignored, which is ≤3% at all current densities and is ≤1% when the density is ≥800 mA·cm^−2^. Consequently, the external humidification on the anode can be removed.
(3) Increasing the back pressure leads to an increase in water content in PEM and an increase in oxygen concentration in CL, which results in a decrease in impedance and an increase in performance at low/no humidification. Therefore, increasing the back pressure is beneficial to achieve self-humidification.
(4) It is more difficult for the stack to achieve self-humidification at higher temperatures because the sulfonate group tends to lose water. However, increasing back pressure can compensate for the negative effect of increasing temperature. Self-humidification can be achieved for the stack operated at 60–90 °C with a suitable back pressure in the range of 100–200 kPa.
(5) It is observed that with an increase in current density, there is a gradual convergence and crossing of the voltage at RHa/RHc = 20%/40% with that at RHa/RHc = 20%/80%, and the crossover point, even for RHa/RHc = 0%, occurs at 2000 mA·cm^−2^, 60 °C, and 200 kPa. Below the current density of the crossover point, the stack’s performance is inferior at lower RH due to membrane unsaturation. Conversely, the stack’s performance is inferior at higher RH due to flooding. Furthermore, the crossover point appears only with the achievement of successful self-humidification, and the current density of the point decreases with increasing pressure and decreasing temperature.

## Figures and Tables

**Table 1:** The parameters of geometric model.

Geometric Parameters	Values (mm)
BP thickness	0.5
Cathode channel length/width/height	120.1/0.8/0.4
Anode channel length/width/height	41.6/0.7/0.3
Anode/cathode rib width	0.5/1
GDL thickness	0.16
MPL thickness	0.03
Cathode/anode CL thickness	0.009/0.006
MEM thickness	0.008

**Table 2:** Physical parameters and boundary conditions [10,11].

	Value
Open-circuit voltage (V)	1.15
Temperature (K)	353.15
Anode/cathode outlet pressure (kPa)	150/150
Anode/cathode stoichiometry	2/1.6
Active area (cm^2^)	49.6614
GDL/MPL/CL contact angle (deg)	130/140/120
GDL/MPL/CL porosity	0.7/0.6/0.5
GDL/MPL/CL/PEM permeability(m^2^)	8 × 10^−12^/5 × 10^−13^/3 × 10^−14^/1 × 10^−18^/0
GDL/MPL/CL density (kg·m^−3^)	440/440/1000
GDL/MPL/CL specific heat capacity (J·kg^−1^·K^−1^)	710/710/3300
GDL/MPL/CL thermal conductivity (W·m^−1^·K^−1^)	1.7/1.7/8
GDL/MPL/CL electrical conductivity (S·m^−1^)	5000/5000/1000
Surface/volume ratio in CL (m^−1^)	200,000
PEM equivalent mass (kg·kmol^−1^)	1100
Anode/cathode exchange current density (mA·cm^−2^)	10,000/10

**Table 3:** Grid independence test.

	Grid Number	Current Density (mA·cm^−2^)	Voltage (V)	Calculated Time (h)
Case 1	8,243,664	2000	0.612416	60
Case 2	10,991,552	2000	0.613369	72
Case 3	13,739,440	2000	0.613764	84
Case 4	18,985,408	2000	0.613349	144

**Table 4:** The test cases of the self-humidification experiment.

Temperature (°C)	Back Pressure (Gauge Pressure, kPa)			
	Anode		Cathode	
60	200		200	
70	200		200	
80	0		0	
	100		100	
	150		150	
	200		200	
90	200		200	
	Flux	RH (%)		
Anode	1.6 SLPM	0	20	20
Cathode	2 SLPM	0	40	80

**Table 5:** The crossover points for different working conditions.

Temperature (°C)	Pressure (kPa)	Self-Humidification (Yes or No)	Current Density of Crossover Point (mA·cm^−2^)
	An/Ca		
60	200/200	Yes	700 (2000 at RH = 0%)
70	200/200	Yes	1100
80	0/0	No	No cross
	100/100	Yes	No cross
	150/150	Yes	2400
	200/200	Yes	1800
90	200/200	No	No cross

## Data Availability

The data presented in this study are available on request from the corresponding author.

## Nomenclature

*c_p_*	constant pressure specific heat, J mol^−1^ K^−1^	*φ*	potential, V
*D_λ_*	diffusivity, m^2^ s^−1^	*χ*	velocity ratio of liquid water to gas in the flow channel
*D_i_*	multi-component diffusivity, m^2^ s^−1^		
*F*	Farady’s constant, 96,485 C mol^−1^		
*EW*	the equivalent mass of PEM, g mol^−1^	Superscripts	
*i*	current density, A m^−2^	*eff*	effective
*k^eff^*	thermal conductivity, W m^−1^ K^−1^	*ref*	reference
*k_ion_*	proton conductivity, S m^−1^	*T*	thermal
*k_ele_*	electron conductivity, S m^−1^		
*M*	molecular weight, kg mol^−1^	Subscripts	
*n*	number	*a*	anode
*n*_d_	electro-osmotic drag coefficient	*c*	condensation
*p*	pressure, Pa	*ca*	cathode
*R*	ideal gas constant, 8.314 J mol^−1^ K^−1^	*Cl*	catalyst layer
*RH*	relative humidity	d	electro-osmotic drag
*S*	source term	*d*	dissolved water
*s*	liquid water saturation	*e*	evaporation
*T*	temperature, K	*ele*	electron
* $\vec{u}$ *	velocity, m s^−1^	*eq*	equilibrium
		*g*	gaseous water
Greek		*i*	species *i*
*κ*	permeability, m^2^	*ion*	ion
*λ*	water content	*j*	species *j*
*μ*	viscosity, Pa s	*k*	species *k*
*ρ*	density, kg m^−3^	*l*	liquid water
*ε*	porosity	*liq*	liquid water
*δ*	thickness, m	*mem*	membrane
*γ_c_*	condensation rate coefficient	*mw*	membrane water
*γ_e_*	evaporation rate coefficient	*sat*	saturation
*γ*	exchange rate coefficient	*λ*	membrane water

## References

[1] Jiao K., Xuan J., Du Q., Bao Z., Xie B., Wang B., Zhao Y., Fan L., Wang H., Hou Z. (2021). Designing the next Generation of Proton-Exchange Membrane Fuel Cells. Nature.

[2] Elwan H.A., Mamlouk M., Scott K. (2021). A Review of Proton Exchange Membranes Based on Protic Ionic Liquid/Polymer Blends for Polymer Electrolyte Membrane Fuel Cells. J. Power Sources.

[3] Sun C., Negro E., Vezzù K., Pagot G., Cavinato G., Nale A., Herve Bang Y., Di Noto V. (2019). Hybrid Inorganic-Organic Proton-Conducting Membranes Based on SPEEK Doped with WO3 Nanoparticles for Application in Vanadium Redox Flow Batteries. Electrochim. Acta.

[4] Kongkanand A., Mathias M.F. (2016). The Priority and Challenge of High-Power Performance of Low-Platinum Proton-Exchange Membrane Fuel Cells. J. Phys. Chem. Lett..

[5] Sharaf O.Z., Orhan M.F. (2014). An Overview of Fuel Cell Technology: Fundamentals and Applications. Renew. Sustain. Energy Rev..

[6] Shao Y., Xu L., Xu L., Zhang X., Wang Z., Zhao G., Hu Z., Li J., Ouyang M. (2023). Water Management Issues during Load Cycling under High Temperature and Low Humidity Conditions Relevant for Heavy-Duty Applications of PEMFC. eTransportation.

[7] Kim J.H., Cho E.A., Jang J.H., Kim H.J., Lim T.H., Oh I.H., Ko J.J., Oh S.C. (2010). Effects of Cathode Inlet Relative Humidity on PEMFC Durability during Startup–Shutdown Cycling. J. Electrochem. Soc..

[8] Chang Y., Qin Y., Yin Y., Zhang J., Li X. (2018). Humidification Strategy for Polymer Electrolyte Membrane Fuel Cells—A Review. Appl. Energy.

[9] Mirfarsi S.H., Parnian M.J., Rowshanzamir S. (2020). Self-Humidifying Proton Exchange Membranes for Fuel Cell Applications: Advances and Challenges. Processes.

[10] Park H. (2014). Effect of the Hydrophilic and Hydrophobic Characteristics of the Gas Diffusion Medium on Polymer Electrolyte Fuel Cell Performance under Non-Humidification Condition. Energy Convers. Manag..

[11] Kong I.M., Choi J.W., Kim S.I., Lee E.S., Kim M.S. (2015). Experimental Study on the Self-Humidification Effect in Proton Exchange Membrane Fuel Cells Containing Double Gas Diffusion Backing Layer. Appl. Energy.

[12] Lin R., Chen L., Zheng T., Tang S., Yu X., Dong M., Hao Z. (2021). Interfacial Water Management of Gradient Microporous Layer for Self-Humidifying Proton Exchange Membrane Fuel Cells. Int. J. Heat Mass Transf..

[13] Koh B.-S., Yoo J.-H., Jang E.-K., Jothi V.R., Jung C.-Y., Yi S.C. (2018). Fabrication of Highly Effective Self-Humidifying Membrane Electrode Assembly for Proton Exchange Membrane Fuel Cells via Electrostatic Spray Deposition. Electrochem. Commun..

[14] Liu J., Yin Y., Zhang J., Zhang T., Zhang X., Chen H. (2021). Mechanical Degradation of Catalyst Layer under Accelerated Relative Humidity Cycling in a Polymer Electrolyte Membrane Fuel Cell. J. Power Sources.

[15] Oh K., Kwon O., Son B., Lee D.H., Shanmugam S. (2019). Nafion-Sulfonated Silica Composite Membrane for Proton Exchange Membrane Fuel Cells under Operating Low Humidity Condition. J. Membr. Sci..

[16] Ren H., Meng X., Lin Y., Shao Z. (2022). Structural Stability of Catalyst Ink and Its Effects on the Catalyst Layer Microstructure and Fuel Cell Performance. J. Power Sources.

[17] Cha D., Jeon S.W., Yang W., Kim D., Kim Y. (2018). Comparative Performance Evaluation of Self-Humidifying PEMFCs with Short-Side-Chain and Long-Side-Chain Membranes under Various Operating Conditions. Energy.

[18] Li Y., Zhou Z., Liu X., Wu W.-T. (2019). Modeling of PEM Fuel Cell with Thin MEA under Low Humidity Operating Condition. Appl. Energy.

[19] Huang H., Xu S., Zhang L., Fan J., Li H., Wang H. (2021). A Self-Humidifying Proton Exchange Membrane Embedded with Phosphonic Acid-Functionalized Mesoporous Silica Nanoparticles That Has Excellent Dispersion and Water Retention. Sustain. Energy Fuels.

[20] Xie Z., Tian L., Zhang W., Ma Q., Xing L., Xu Q., Khotseng L., Su H. (2021). Enhanced Low-Humidity Performance of Proton Exchange Membrane Fuel Cell by Incorporating Phosphoric Acid-Loaded Covalent Organic Framework in Anode Catalyst Layer. Int. J. Hydrogen Energy.

[21] Shin S., Maiyalagan T., Jothi V.R., Jung C.Y., Yi S.C. (2021). Numerical Analysis on Transport Properties of Self-Humidifying Dual Catalyst Layer via 3-D Reconstruction Technique. Int. J. Hydrogen Energy.

[22] Hou S., Wang H., Ren J., Yao C., Shi L., Liao S. (2022). Enhanced Low-Humidity Performance of Proton-Exchange Membrane Fuel Cell by Introducing Hydrophilic CNTs in Membrane Electrode Assembly. Prog. Nat. Sci. Mater. Int..

[23] Angayarkanni R., Ganesan A., Dhelipan M., Karthikeyan S., Mani N., Thiyagarajan P. (2022). Self-Humidified Operation of a PEM Fuel Cell Using a Novel Silica Composite Coating Method. Int. J. Hydrogen Energy.

[24] Ren G., Qu Z., Wang X., Zhang J. (2022). Liquid Water Transport and Mechanical Performance of Electrospun Gas Diffusion Layers. Int. J. Green Energy.

[25] Li X., Zhang Z., Xie Z., Guo X., Yang T., Li Z., Tu M., Rao H. (2022). High Performance and Self-Humidifying of Novel Cross-Linked and Nanocomposite Proton Exchange Membranes Based on Sulfonated Polysulfone. Nanomaterials.

[26] Tong G., Xu X., Yuan Q., Yang Y., Tang W., Sun X. (2021). Simulation Study of Proton Exchange Membrane Fuel Cell Cross-convection Self-humidifying Flow Channel. Int. J. Energy Res..

[27] Wang Z., Zhan Z., Tan J., Pan M. (2019). Water transport law of fuel cell membranes at different current densities. Chin. Sci. Bull..

[28] Zhang R., Yang B., Lei X., Ming P., Li B., Lei Y., Yang D., Zhang C. (2022). Droplets Dynamics Theory and Micro-Flow Field Experiments of Improving Self-Humidifying Feature and Maximum Power Density in Fuel Cells. Chem. Eng. J..

[29] Lian Y., You C., Zhu Z., Zhu X., Li X., Zhou W. (2022). Preparation and Performance of a Self-Humidifying Fuel Cell Using a Fiber Sintered Sheet as Flow Field. J. Power Sources.

[30] Lian Y., Zhu Z., You C., Lin L., Lin F., Lin L., Huang Y., Zhou W. (2023). Structural Optimization of Fiber Porous Self-Humidifying Flow Field Plates Applied to Proton Exchange Membrane Fuel Cells. Energy.

[31] Fan L., Zhang G., Jiao K. (2017). Characteristics of PEMFC Operating at High Current Density with Low External Humidification. Energy Convers. Manag..

[32] Zhao X., Xu L., Fang C., Jiang H., Li J., Ouyang M. (2018). Study on Voltage Clamping and Self-Humidification Effects of Pem Fuel Cell System with Dual Recirculation Based on Orthogonal Test Method. Int. J. Hydrogen Energy.

[33] Shao Y., Xu L., Zhao X., Li J., Hu Z., Fang C., Hu J., Guo D., Ouyang M. (2020). Comparison of Self-Humidification Effect on Polymer Electrolyte Membrane Fuel Cell with Anodic and Cathodic Exhaust Gas Recirculation. Int. J. Hydrogen Energy.

[34] Zhang Q., Tong Z., Tong S. (2020). Effect of Cathode Recirculation on High Potential Limitation and Self-Humidification of Hydrogen Fuel Cell System. J. Power Sources.

[35] Zhang Q., Tong Z., Tong S., Cheng Z. (2021). Self-Humidifying Effect of Air Self-Circulation System for Proton Exchange Membrane Fuel Cell Engines. Renew. Energy.

[36] Liu Z., Xu S., Guo S. (2022). High-Potential Control for Durability Improvement of the Vehicle Fuel Cell System Based on Oxygen Partial Pressure Regulation under Low-Load Conditions. Int. J. Hydrogen Energy.

[37] Li Q., Gong J., Peng S., Lu S., Sui P.-C., Djilali N., Xiang Y. (2016). Theoretical Design Strategies of Bipolar Membrane Fuel Cell with Enhanced Self-Humidification Behavior. J. Power Sources.

[38] Heo P., Kim M., Ko H., Nam S.Y., Kim K. (2021). Self-Humidifying Membrane for High-Performance Fuel Cells Operating at Harsh Conditions: Heterojunction of Proton and Anion Exchange Membranes Composed of Acceptor-Doped SnP_2_O_7_ Composites. Membranes.

[39] Wang J., Wang B., Tongsh C., Miao T., Cheng P., Wang Z., Du Q., Jiao K. (2022). Combining Proton and Anion Exchange Membrane Fuel Cells for Enhancing the Overall Performance and Self-Humidification. Chem. Eng. J..

[40] Corda G., Fontanesi S., d’Adamo A. (2022). Methodology for PEMFC CFD Simulation Including the Effect of Porous Parts Compression. Int. J. Hydrogen Energy.

[41] Jiao K., Li X. (2011). Water Transport in Polymer Electrolyte Membrane Fuel Cells. Prog. Energy Combust. Sci..

[42] Li S., Sundén B. (2018). Effects of Gas Diffusion Layer Deformation on the Transport Phenomena and Performance of PEM Fuel Cells with Interdigitated Flow Fields. Int. J. Hydrogen Energy.

[43] Jiang P., Zhan Z., Zhang D., Wang C., Zhang H., Pan M. (2022). Two-Dimensional Simulation of the Freezing Characteristics in PEMFCs during Cold Start Considering Ice Crystallization Kinetics. Polymers.

[44] Zhu R., Zhan Z., Zhang H., Du Q., Chen X., Xiang X., Wen X., Pan M. (2023). Effects of Cathode GDL Gradient Porosity Distribution along the Flow Channel Direction on Gas–Liquid Transport and Performance of PEMFC. Polymers.

[45] Ji M., Wei Z. (2009). A Review of Water Management in Polymer Electrolyte Membrane Fuel Cells. Energies.

[46] Wallnöfer-Ogris E., Pertl P., Trattner A. (2020). Quasi-Stationary UI-Characteristic Model of a PEM Fuel Cell–Evaluating the Option of Self-Humidifying Operation. Int. J. Hydrogen Energy.

[47] He X., Zhan Z., Zhang H., Shua L., Sui P.C., Xu L. (2017). The Optimal Control of PEM Fuel Cell Operating at Large Current Density Based on Water Balance. J. Eng. Thermophys..

[48] Tang X., Zhang Y., Xu S. (2023). Experimental Study of PEM Fuel Cell Temperature Characteristic and Corresponding Automated Optimal Temperature Calibration Model. Energy.

[49] Luo Z., Chang Z., Zhang Y., Liu Z., Li J. (2010). Electro-Osmotic Drag Coefficient and Proton Conductivity in Nafion® Membrane for PEMFC. Int. J. Hydrogen Energy.

[50] Li Y., Pei P., Wu Z., Ren P., Jia X., Chen D., Huang S. (2018). Approaches to Avoid Flooding in Association with Pressure Drop in Proton Exchange Membrane Fuel Cells. Appl. Energy.

